# Molecular-based allergy diagnostics in the real world: evidence for a cost explosion and an impact vacuum

**DOI:** 10.1186/2045-7022-5-S3-O3

**Published:** 2015-03-30

**Authors:** Niall Conlon, Lisa Devlin, Cathal Steele, Hiba Shendi, Matthew Doré, John Thompson, Alison Donnelly, J David M Edgar

**Affiliations:** 1 Centre for Infection and Immunity, Queen's University Belfast, Ireland; 2 Department of Immunology, Royal Victoria Hospital, Belfast, Ireland

## Background

Molecular-based allergy (MA) diagnostics are used to identify patient sensitization to individual allergen components. Despite a recent consensus statement, the clinical utility of MA diagnostics in routine practice remains unclear.

## Aim

To review the cost and usage patterns of MA diagnostics at a regional level and to estimate the clinical value of such testing strategies.

## Methods

Laboratory data on Northern Ireland MA diagnostics ordering between 2010 and 2013 was obtained. Data specific to Regional Immunology Service patients from 2013 was further interrogated. A retrospective review of case records from adults (n=70) and children (n=53) was carried out to determine the impact of MA test results.

## Results

**Figure 1:**
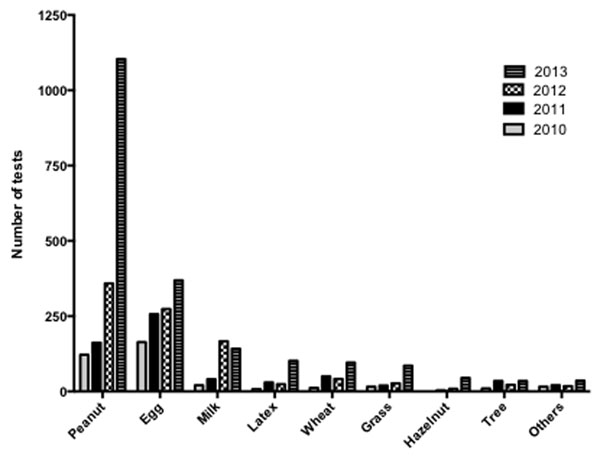


**Figure 2:**
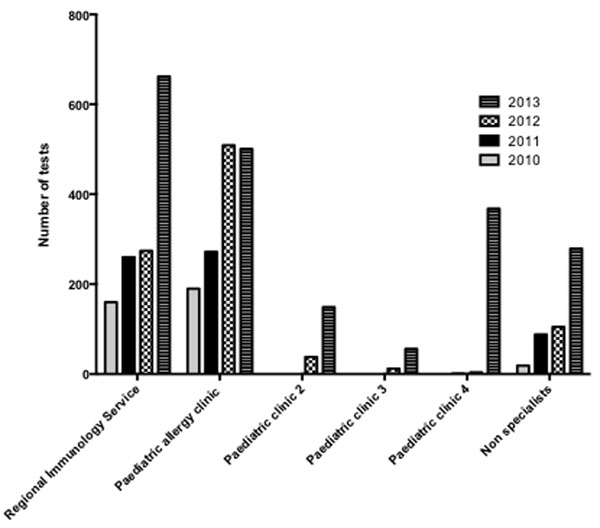


MA testing has increased annually since its introduction in 2010. Spending on MA diagnostics exceeded £20,000 in 2013. Increases in test numbers were observed across a range of allergen groups during the time period and suggests routine ordering (Figure 1). Analysis of request sources indicated a surge in test numbers across 5 key user hospital units (Figure 2). Additionally, a marked increase in MA diagnostics orders from non-specialist sources was noted.

Use of MA diagnostics introduced a mean increase in cost of 156% in adults (range 11%-900%) and 197% in children (range 9%-600%)(Table 1). Review of individual cases indicated that MA diagnostics had no specific impact on diagnosis or management in 78.6% of adults and 83.0% of children.

**Table 1:** 

	Adults (n=70)	Children (n=53)
**Mean age (sd)**	31.9 (12.3)	9.2 (4.2)

**% cost increase with MA (range)**	156 (11-900)	197 (9-600)

**MA test results altered management, n(%)**	6 (8.6)	7 (13.2)

**MA test results provided useful diagnostic support, n(%)**	9 (12.8)	2 (3.7)

**MA test results had no impact on diagnosis or management, n(%)**	55 (78.6)	44 (83)

## Discussion

This study suggests that the use of MA diagnostics is entering routine clinical practice and is associated with a cost premium. The minimal clinical impact of these testing modalities argues for the establishment of demand management programs in resource-constrained environments.

